# Circulating Serum miRNA-205 as a Diagnostic Biomarker for Ototoxicity in Mice Treated with Aminoglycoside Antibiotics

**DOI:** 10.3390/ijms19092836

**Published:** 2018-09-19

**Authors:** Sun Hee Lee, Hyun Mi Ju, Jin Sil Choi, Yeji Ahn, Suhun Lee, Young Joon Seo

**Affiliations:** 1 Laboratory of Smile Snail, Yonsei University Wonju College of Medicine, Wonju, Gangwon-do 26426, Korea; shl7547@gmail.com (S.H.L.); skdi1082@naver.com (H.M.J.); towtowtow92@naver.com (J.S.C.); yehzzih@naver.com (Y.A.); tngns6049@daum.net (S.L.); 2 Department of Otorhinolaryngology, Yonsei University Wonju College of Medicine, Wonju, Gangwon-do 26426, Korea

**Keywords:** ototoxicity, microRNA, hearing loss, serum

## Abstract

Background: To confirm levels and detection timing of circulating microRNAs (miRNAs) in the serum of a mouse model for diagnosis of ototoxicity, circulating miR-205 in the serum was evaluated to reflect damages in the cochlear microstructure and compared to a kidney injury model. Method: A microarray for miRNAs in the serum was performed to assess the ototoxic effects of kanamycin-furosemide. Changes in the levels for the selected miRNAs (miR-205, miR-183, and miR-103) were compared in the serum and microstructures of the cochlea (stria vascularis, organ of Corti, and modiolus) between the ototoxicity and normal mouse groups. An acute kidney injury (AKI) mouse model was used to assess changes in miR-205 levels in the kidney by ototoxic drugs. Results: In the mouse model for ototoxicity, the serum levels of circulating miR-205 peaked on day 3 and were sustained from days 7–14. Furthermore, miR-205 expression was highly expressed in the organ of Corti at day 5, continued to be expressed in the modiolus at high levels until day 14, and was finally also in the stria vascularis. The serum miR-205 in the AKI mice did not change significantly compared to the normal group. Conclusions Circulating miR-205 from the cochlea, after ototoxic damage, migrates through the blood vessels to organs, which is then finally found in blood. In conditions of hearing impairment with ototoxic medications, detection of circulating miR-205 in the blood can be used to determine the extent of hearing loss. In the future, inner ear damage can be identified by simply performing a blood test before the hearing impairment due to ototoxic drugs.

## 1. Introduction

An estimated four million patients in the United States are treated with aminoglycoside antibiotics annually [1]. Aminoglycoside causes hearing loss in up to 10% of patients receiving these drugs intravenously [2]. Ototoxicity is typically associated with bilateral high-frequency sensorineural hearing loss and tinnitus. Hearing loss can be temporary but is usually irreversible with most agents. The usual time of onset is often unpredictable and marked hearing loss can occur even after a single dose. Additionally, hearing loss may not manifest until several weeks or months after completion of antibiotic or antineoplastic therapy. Clinicians could identify patients affected by ototoxicity drugs only after complaints because of the delayed onset of hearing loss. Therapeutic drug monitoring (TDM) has been the only prognostic prediction method during aminoglycoside treatment to achieve efficient serum concentrations of the drug and minimize the risk of ototoxicity [3]. Early detection would allow the health care provider to examine potential treatment alternatives prior to onset of hearing impairment that can affect communicative ability.

MicroRNAs (miRNAs) are potent modulators of gene expression and are involved in almost every primary biological process including proliferation, apoptosis, differentiation, and organogenesis [4,5,6,7]. Lawrie et al. (2008) reported the first evidence demonstrating that miRNAs could be reliably detected in serum [8], and the number of circulating miRNAs detected ranged from 200 to over 450 depending on the fluid investigated [6]. Detection of circulating miRNAs, which is a major challenge in modern medicine, can potentially provide clinical utility for miRNAs as diagnostic tools. The ability to identify a disease in its early phases would allow for rapid intervention and consequently prevent disease progression. Rosetta Genomics has already commercially launched a miRView lung assay that consists of a panel of eight miRNAs for the identification of primary lung tumors [9]. An alternative of Carcino-Embryonic Antigen (CEA) and Carcinoma Antigen-15-3 (CA-15-3) was proposed by Heneghan et al. (2010), who identified patients with breast cancer with a sensitivity and specificity of 87.7% and 91%, respectively, by measuring the circulating levels of miR-195 [10].

In the current study, the expressions of circulating miRNAs were analyzed in peripheral blood serum of a mouse model for ototoxicity with kanamycin and furosemide in comparison with healthy subjects to determine miRNAs that can be used as a diagnostic biomarker. We used miR-205 to investigate the correlation of circulating miRNA-205 in the serum with locally expressed miRNAs (miR-183 and miR-205) of the cochlea, and determined the best timing for detecting miRNA-205.

## 2. Results

### 2.1. The Mice with Ototoxicity Induced by the “One-Shot” Method Are Effective Ototoxicity Models without Kidney Impairment

**Figure 1:**
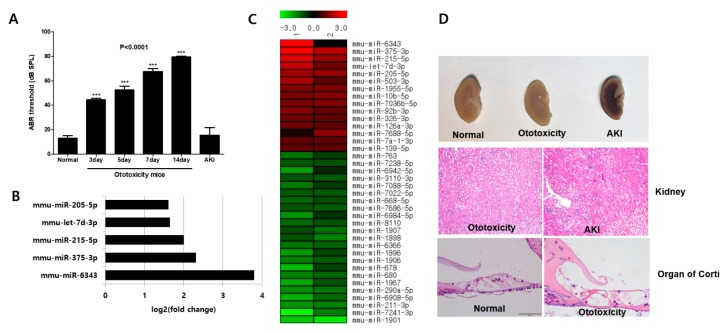
Auditory brainstem response (ABR) threshold test and global miRNA expression profiles in ototoxicity-induced mice. (**A**) The ABR threshold shifted on days 3, 5, 7, and 14 in the ototoxicity mice group gradually increased than control group. The hearing loss was not observed in the control acute kidney injury (AKI) mouse model. *** *p* < 0.0001. (**B**) Microarray analysis of circulating miRNAs revealed 5 major miRNAs that increased in the serum of the ototoxicity-induced mouse model compared to the control. (**C**) Cluster heat maps illustrated clustering of circulating miRNA in the ototoxicity-induced mice. Hierarchical clustering analysis between groups of ototoxicity-induced and normal mice represent higher and lower relative expression (red and green, respectively), which were based on differential expression of 38 significantly altered miRNAs. (**D**) Hematoxylin and eosin stained tissue sections from the organ of Corti and Kidney. The AKI model showed an infarction in the outer medullar but was normal in the ototoxicity mouse model. Damage to the inner and outer hair cells and disappearance of supporting cells was observed in the organ of Corti of ototoxicity-induced mice.

We previously studied the ototoxicity-induced mouse model using a combination of kanamycin and furosemide, which we called the “one-shot” method [11]. We preferentially attempted to identify key molecules involved in the ototoxicity-inducing mechanism. To characterize the ototoxicity model, we compared it with the acute kidney injury (AKI) model. In Figure 1A, the auditory brainstem response (ABR) test was used to determine hearing loss on days 0, 3, 5, 7, and 14. The ABR test revealed that hearing decreased gradually. The threshold for normal mice was 17 dB. In contrast, the threshold for the ototoxic group increased significantly to 45 dB by day 3, and 80 dB by day 14. These thresholds were about 4-fold higher than the AKI group. The mice with AKI did not survive over 36 h, so we were only able to obtain data for hearing on day 1. To identify the main molecule involved in ototoxicity, we performed a microarray analysis with 2 normal and 4 mice with ototoxicity (Figure 1D). We confirmed the accuracy of ototoxicity and AKI mouse model by hematoxylin and eosin staining. The damage in the inner ear was marked in the organ of Corti. It showed that the outer hair cells were completely collapsed, and the tectorial membrane was damaged. In the kidney section, the AKI model had an infarction in the outer medulla, but not in the normal and ototoxicity group. Kidney damage was prominent in the tubules of the outer medulla. All tubules were loose and irregular.

We identified 3165 miRNAs in the ototoxicity-induced mice and selected 38 miRNAs that showed significant expression. Fifteen and 23 miRNAs had increased and decreased expressions, respectively, in the ototoxicity-induced mice. MiRNAs that increased or decreased more than 3-fold in the heat map were marked in red and green, respectively (Figure 1C). Among them, the five miRNAs that increased the most had fold changes ranging from 1.6 to 3.8-fold (Figure 1B). With these profiles, we then selected target miRNAs by comparison to previous studies. Tal Elkan-Miller et al. (2011) previously confirmed that miR-205 was expressed only in the cochlea via miRNA profiling of the vestibular and cochlear end-organs [12]. Furthermore, miR-205 was the only overlapping miRNA between our profile and the references. In addition, Friedman et al. (2009) revealed that miRNAs play an essential role in the development of the cochlea [13]. Various studies have demonstrated that miR-183 is locally expressed in the inner ear [13,14,15]; therefore, we chose miR-183 expression in sensory epithelia as a control. The levels of miR-103 were also selected as a control in the serum. We therefore hypothesized that miR-205 in the serum plays a role in the ototoxicity-inducing mechanism and therefore selected it as a target circulating miRNA.

### 2.2. Expression of miR-205 in the Serum Is Specific to the Ototoxicity-Induced Mouse Model

**Figure 2:**
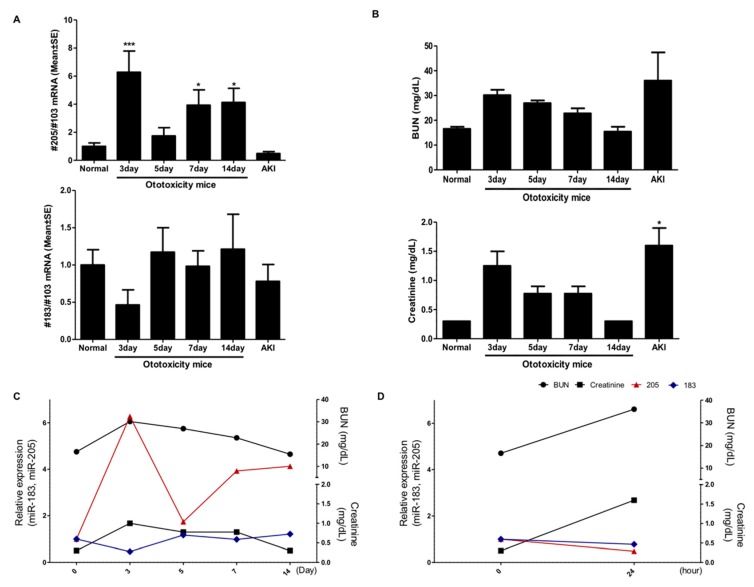
Comparison of expression levels of miR-205 and miR183 in the serum. (**A**) The expression of miR-205 and miR183 in the serum was examined using qRT-PCR. MiR-205 was significantly elevated in the serum compared to the control group. In the serum, miR-205 expression peaked in the ototoxicity-induced mice at day 3, and the levels increased more than 4-fold between days 7 and 14. There were no differences of miR-183 levels in the serum. (**B**) The blood urea nitrogen and creatinine levels were elevated by day 3, and then recovered gradually, but remained elevated in the AKI mouse model. * *p* < 0.01 and *** *p* < 0.0001. (**C**) The levels of miR-205, miR-183, Blood urea nitrogen (BUN), and creatinine are shown in the line graph for 14 days in the ototoxicity-induced mouse model. (**D**) represents for 24 h in the AKI mice model.

We identified the expression of miR-205 and miR-183 using qRT-PCR (Figure 2). Because aminoglycosides and furosemide could injury the kidney, we evaluated the levels of miR-205 and miR-183 with BUN (Blood urea nitrogen) and creatinine levels compared to the AKI model. We observed that miR-205 increased over 6-fold compared to wild-type and AKI model mice by day 3, and a 4-fold increase was maintained from days 7 to 14 (Figure 2A). In contrast, miR-183 levels were not significantly different compared to wild-type and AKI model mice. In Figure 2B, we determined whether damaged ears affected kidney function. BUN and creatinine levels of AKI mice were very high, therefore demonstrating that kidney function was affected. On day 3, BUN and creatinine levels in the ototoxicity group were higher than normal (not significantly), but gradually decreased and stabilized by day 14. Ototoxicity could affect kidney function at the beginning of the ototoxic induction, but kidney function was slowly restored over time. In the kidney injury mouse model, though the creatinine levels increased significantly, the circulating miR-205 levels were not increased in the serum. Therefore, increasing levels of miR-205 until day 14 could reflect changes to the cochlea by ototoxic drugs (Figure 2C,D).

### 2.3. Circulating miR-205 Plays an Important Role in Ototoxicity

**Figure 3:**
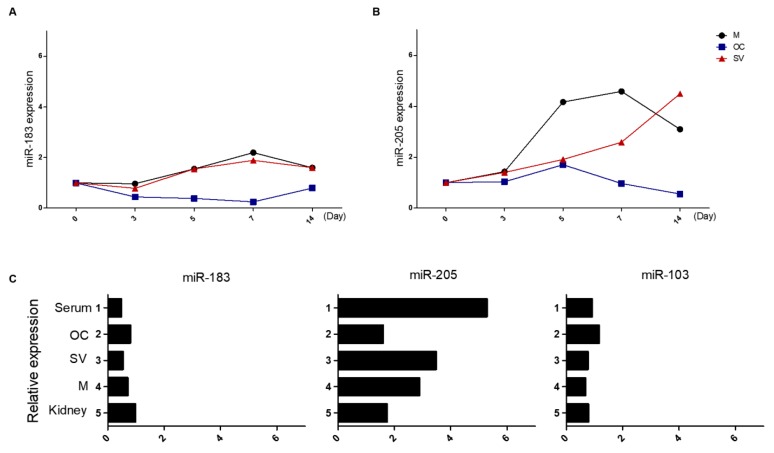
Comparison of miR-205 and miR183 expression levels in the mouse ear. The expressions of miR-183 (**A**) and miRNA-205 (**B**) in the stria vascularis (SV), organ of Corti (OC), and modiolus (M) were examined using qRT-PCR. The expression of miR-205 in the stria vascularis in the ototoxicity-induced mouse group gradually increased and was highest at day 14. (**C**) The levels of miR-183, miR-205, and miR-103 in the serum at day 14 after the induction of ototoxicity was compared with levels in the microstructures of the cochlea and kidney. Levels of miR-205 was highest in the serum, and levels in the stria vascularis were also increased. No differences were observed for the levels of miR-183 and miR-103. *p* < 0.0001.

To confirm that the circulating miR-205 originated from the inner ear, we dissected the cochlea and separated it into three components; the organ of Corti, stria vascularis, and modiolus. Levels of miR-183 were slightly increased on day 7 in the stria vascularis and modiolus, while miR-205 was highly expressed in a unique manner. In the organ of Corti, miR-205 was slightly increased only on day 5, but its expression was very high in modiolus, and then decreased by day 14. Interestingly, miR-205 expression in the stria vascularis increased gradually and peaked at day 14. These results suggested that miR-205 can migrate through the stria vascularis. Thus, we compared levels of miR183, miR-205, and miR103 in the serum of mice with ototoxicity to levels in the cochlea and kidney using qRT-PCR (Figure 3). The expression of miR-205 was highest in the serum and secondly in the stria vascularis. Furthermore, there were no differences in expression between miR-183 and miR-103.

Taken together, we hypothesized that miR-205 would extravasate through the stria vascularis during circulation when ototoxicity was induced. In the early stages of ototoxicity induction, miR-205 was expressed in the organ of Corti and then migrated over time via the stria vascularis and modiolus. Although miR-183 seems to be involved in the ototoxicity induction mechanism, it is not enough to explain the molecular mechanism of ototoxicity with aminoglycosides and furosemide, since there were no significant changes in the cochlea.

### 2.4. Circulating miR-205 is a Potential Target Molecule for Ototoxicity-Induced Hearing Loss

We sought to determine whether expression of circulating miR-205 was consistent with the damaged inner ear. Through in situ hybridization, miR-205 was highly expressed in the outer hair cells, stria vascularis, and spiral ganglion. These results suggest that the ototoxicity mechanism was closely related to miR-205 expression. It is clear that miR-205 expression is directly involved in the increase of ototoxicity.

## 3. Discussion

miRNAs are endogenous small RNAs that are 21–25 nucleotides long and can pair sequences in the 3′ untranslated region in mRNAs of protein-coding genes to downregulate their expression [4]. The presence of miRNAs in various body fluids has recently been reported. Accumulating evidence suggests the usefulness of circulating miRNAs as stable blood-based biomarkers for the detection of various cancers [6,7,8]. Because miRNA profiles appear to be cell-specific, the identification of a profile for increased miRNAs in circulating blood may help determine the type of injury [10]. Our data demonstrated that serum concentration of miR-205, which was produced exclusively in the injured cochlea, increased in the aminoglycoside and furosemide ototoxicity-induced mouse model. The serum levels of miR-205 after the induction of ototoxicity was sustained for 14 days, and this was well correlated with the levels in the stria vascularis. Thus, our results clearly support the hypothesis that miRNAs may leak out of injured cells into the circulating blood and thereby serve as markers for identifying the type of injury.

We developed a mouse model that can effectively induce ototoxicity. In the “one-shot” method, kanamycin and furosemide can be administered together to produce a ototoxicity-induced mouse model with only one injection within a shorter period of time compared to conventional methods [11]. The precise mechanisms by which kanamycin and furosemide induce ototoxicity are not known. According to many studies, cytotoxicity mechanisms in the inner ear induced by kanamycin are mostly associated with apoptosis. However, cytotoxicity induced by high concentrations of kanamycin was observed to exhibit morphological features of necrosis [16]. Thus, this study suggests that the establishment of an ototoxicity model based on combination treatments with low concentrations of kanamycin and furosemide may be associated with a novel mechanism that is different from conventional cell death mechanisms. No other organ damage was found to be irreversible due to the ototoxic drugs. The organs that can be most damaged by this drug are the kidneys, which is why we used an AKI mice model. In the renal toxicity model, it was important to use an animal model that have kidneys susceptible to damage without ototoxicity. The AKI mouse model was difficult to care for over 48 h due to a sudden drop in renal function, and it was difficult to confirm the levels of miRNA in the serum for more than 2 weeks as in the ototoxicity-induced mouse model.

Several studies have been reported on miRNAs with ototoxicity models. For example, miR-183 has been shown to be actively expressed in kanamycin-induced ototoxicity [17], and miR-207 was also directly related to radiation-induced apoptosis [18]. However, these miRNAs were expressed around injured tissues. Our results showed changes in circulating miRNA levels in the serum, such as miR-205, via microarray analysis. miR-205 has been demonstrated to induce epithelial-mesenchymal transition under hypoxia conditions [19,20], and has also been shown to induce bladder cancer or corneal disease [21]. Therefore, we confirmed the increase in miR-205 levels via microarray and speculated that this increase was closely related to the induction of ototoxicity. Thus, we ultimately selected miR-205 as a target molecule for the ototoxicity-induced mechanism, and then investigated the miR-205 pathway to determine whether miR-205 specifically affected the ear.

**Figure 4:**
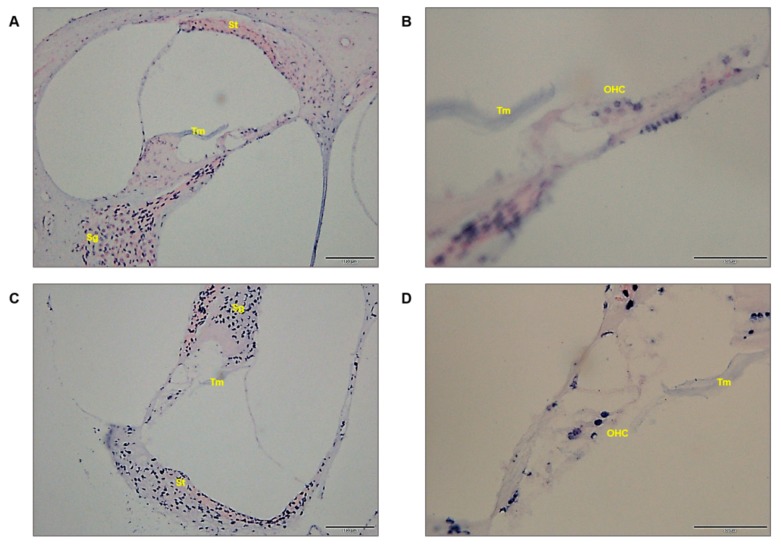
miR-205-specific in situ hybridization analysis. The expression of miR-205 in normal (**A**,**B**) and ototoxicity groups (**C**,**D**). Normal and ototoxicity mouse groups were positively stained in the spiral ganglion and outer hair cells. However, miR-205-positive staining was observed, especially in the stria vascularis of the ototoxicity-induced mouse group. Stria vascularis, St; spiral ganglion, Sg; outer hair cell, OHC; tectorial membrane, Tm. The magnification of (**A**,**C**) is 400×, and the scale bar is 100 μM. (**B**,**D**) represent 600× magnification, and the scale bar is 50 μM.

At the serum level, miR-205 was significantly increased initially, and was about 4-fold higher by day 14 in the ototoxicity-induced mouse model compared to the control group. Furosemide was introduced into the blood vessels to help accumulate kanamycin, maintaining high levels in the cochlea. Combination therapy with a low dose of kanamycin-furosemide is presumed to cause only inner ear damage more effectively. During the early phases of the ototoxicity induction, the high expression of miR-205 in the kidney was present by day 3, but no kidney abnormalities were observed after 14 days. Although the rapid increase in miR-205 on the first 3 days may be due to temporary kidney damage, miR-205 did not increase after 24 h in the AKI animal model. The initial increase in miR-205 may not be correlated with the increase in kidney damage and could have instead originated from other damaged tissues. Alternatively, miR-205 in the injured kidney tissue may have temporarily increased over 24 h and can be detected in the serum by day 3. However, it is clear that levels of miR-205 increased from day 7 to 14 because of damage to the cochlea. In the stria vascularis, which is associated with systemic circulation, levels of miR-205 peaked at 2 weeks, suggesting that miR-205, which was observed on days 7 to 14, may have originated from the ear. To investigate the mechanism of action of the miRNAs, it was necessary to dissect inner ear tissues and identify miRNA expression at each site, which was expected to play an important role in determining the order of damage to inner ear. In Figure 4, miR-205 expression was highly expressed in the organ of Corti at day 5, continued to be expressed in the modiolus at high levels until day 14, and was finally expressed in the stria vascularis. According to studies published so far, when ears begin to be damaged by aminoglycoside antibiotics, hair cells are first affected, and finally the spiral ganglion and stria vascularis are damaged [22].

The miR-205 appears to be initially expressed in the organ of Corti and gradually decreases. The order in which the inner ear is destroyed was consistent with the expression pattern of miR-205.

The miR-183 was not expressed in organ of Corti in our study, and therefore could not be used to explain the ototoxicity mechanisms induced by the kanamycin-furosemide combination. However, some studies showed the increase of miR-183 in the cochlea. According to the review of Ushakov et al. [23], the miR-183 family is the most characterized miRNA cluster in the inner ear. Patel et al. [24] also indicated that the miR-183/Taok1 target pair is likely to play a role for hair cell death in sensorineural hearing loss by acoustic trauma. In our study, we used the combinations of kanamycin and furosemide in one-shot dose. We supposed that miR183 did not increase in our experiments because the dose of the drug was different, and the damaged microstructures of the cochlea were different from the existing references. Aminoglycoside antibiotics such as kanamycin have been reported to act directly on stria vascularis, leading to morphological changes in the vascular wall, and localization to other supporting cells or spiral ligaments [25,26]. Yu et al. showed that the kanamycin made the cell death increased in particular in the stria vascularis, supporting cells and spiral ganglion cells, and then both miR-34a and miR-34c were significantly elevated, as compared to untreated controls. The signaling pathway of disease caused by the aminoglycoside antibiotics is known to be associated with ROS (reactive oxygen species), and miR-183 may be involved in this process [27]. In this study, we have not studied all possible miRNAs. In the future, we will study the mechanisms of miRNA increase and possible miRNAs.

## 4. Materials and Methods

### 4.1. Animal Model

Forty-six male C57BL/6 mice (Central Lab. Animal Inc., Seoul, Korea), including 24 mice for the changes of hearing thresholds after being treated with ototoxic drugs, were used in this experiments as described in a previous study [11]. Animals were housed at a constant temperature (20 ± 2 °C) and humidity level (50–60%) with a 12-hour light and dark cycle with free access to water and food. Shortly, the mouse model for ototoxicity was generated by a single dose of kanamycin (550 mg/kg, SC; Sigma-Aldrich, Oakville, ON, Canada), followed 30 min later with a dose of furosemide (130 mg/kg, IV; Sigma-Aldrich). The left renal pedicle in AKI mice were ligated with 8/0 silk suture (Ethicon, Norderstedt, German) for 25 min [28]. Six mice (*n* = 2 and 4 for control and experimental, respectively) were used for screening the effective circulating miRNAs in the mice model for ototoxicity via microarray analysis. The mice with ototoxicity were followed for 2 weeks, and the serum of each group (*n* = 6) was collected at days 3, 5, 7, and 14, and compared with the no treatment group (control). The serum in the AKI group (*n* = 6) were collected at day 1, because the AKI mice could survive over 2 days.

**Figure 5:**
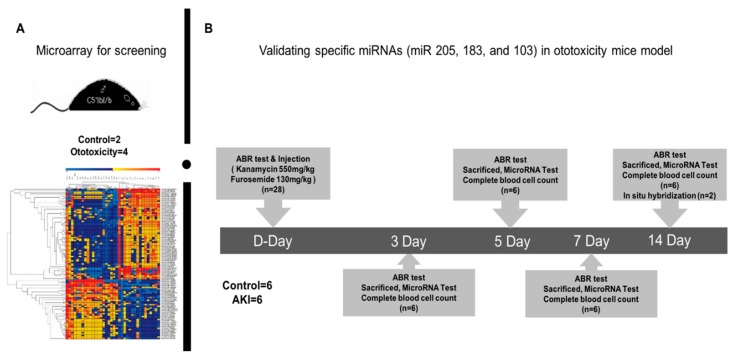
Scheme for verifying specific miRNAs in the ototoxicity-induced mouse model. (**A**) To screen specific miRNAs in ototoxicity-induced mice, 3165 miRNAs were identified, hierarchical clustered, and the expression levels were graphically displayed. The process for establishing the ototoxicity-induced mouse model for 14 days is shown. (**B**) During the 14 days, mice were tested with hearing thresholds by an auditory brainstem response test, and levels of circulating miR-205 and miR-183 compared to miR-103 in the serum and cochlea are shown, as well as the local expression pattern of miR-205 by in situ hybridization.

Normal and ototoxic drug-administrated mice were sacrificed after an auditory brainstem response (ABR) test and serum collection. The temporal bones were removed, and the membrane labyrinth were dissected. We separated the three components of the cochlea into the organ of Corti (OC), modiolus (M), and stria vascularis (SV) under a dissecting microscope. Because of the small amount of tissues for miRNA, the four tissues (two mice) had to be collected and analyzed as a single sample. Finally, 2 control mice with no treatment and 2 mice with ototoxicity at 14 days were used for in situ hybridization analysis (Figure 5).

The animal experiments were approved by the Ethics Committee (Project Identification Code: YWC-160826-1; Approval Date: 26 August 2016) for Animal Experiments of Yonsei University and conducted in accordance with the guidelines of the Institutional Animal Care. All animal experiments were performed in accordance with relevant guidelines and regulations of Yonsei University.

### 4.2. ABR Test for Hearing Thresholds

The thresholds for the ABR test were measured in all the mice. Each animal was gently anaesthetized with an intraperitoneal injection of ketamine (100 mg/kg; Yuhan Corporation, Seoul, Korea) and xylazine (1 mg/kg; Rompun, Korea Bayer, Ansan, Korea) and kept warm using a heating pad. Subdermal needle electrodes were placed at the scalp vertex (non-inverting), posterior bulla (inverting), and lower back (ground) for recording the ABR in the anaesthetized mice. The test stimuli were clicks generated using the BioSigRP (Tucker-Davis Technologies Inc., Alachua, FL, USA). The stimulus intensity decreased gradually in 5-dB steps until the visually discernible ABR waveform disappeared, and the lowest sound level that caused this waveform was defined as the threshold. The scanning time was 10 ms, and 1024 sweeps were averaged with a 300–3000 Hz filtering bandwidth. Threshold shifts were reported as the difference between the pre- and post-test ABR thresholds. Post-test ABR thresholds after kanamycin and furosemide administration were measured at 0, 3, 5, 7, and 14 days.

### 4.3. Blood Collection

The tail of mice was warmed with a light to dilate the blood vessels and wiped with sterilizing cottons containing 70% ethanol. A 1-inch-long 24-gauge needle was used and inserted into 1–2 cm of the distal part of the left tail vein at an angle of approximately 20° under the conscious conditions, and blood (approximately 40 μL/time) was naturally withdrawn by capillary action. After removing the needle, the microcapillary tube containing the blood was centrifuged at 15,000× *g* for 5 min (MC-201, Hitachi, Tokyo, Japan), and plasma was obtained by cutting a border between the supernatant and cell packages using the file. Serum BUN: urease-GLDH method) and creatinine (enzymatic method) concentrations were measured with an autoanalyzer (Accute TBA-40FR, Toshiba Medical Systems, Tochigi, Japan).

### 4.4. Microarray

Total RNA was extracted using the TRIzol Reagent (Invitrogen, Carlsbad, CA USA) according to the manufacturer’s instructions. RNA quality and quantity was assessed by Agilent bioanalyzer 2100 analysis (Agilent Technologies, CA, USA).

Starting with 250 ng of total RNA, the labeling process begins with poly-A tailing of each RNA strand using poly-A polymerase, followed by ligation of biotin-labeled 3DNA dendrimer. Biotinylated RNA strands were hybridized at 48 °C for 18 h on the GeneChip microRNA 4.0 Array (Affymetrix, Santa Clara, CA, USA). The GeneChip microRNA 4.0 Array was washed and stained in the Fluidics Station 450 (Affymetrix, CA, USA). Amplified fluorescence signals by the branched structure of 3DNA dendrimer were scanned using GeneChip Scanner 3000 7G (Affymetrix).

The arrays were analyzed using an Agilent scanner with associated software. The expression levels of miRNAs were calculated with Expression Console 1.4 (Affymetrix, Santa Clara, CA, USA). Relative signal intensities for each miRNA were generated using the Robust Multi-Array Average algorithm. Target prediction were analyzed using the miRBase, miRDB, TargetScan and microRNA.org DB.

### 4.5. In Situ Hybridization Analysis

To identify the expression of miR-205, we used specific miR-205 probes from Exiqon. All experiments were performed according to the miRCURY LNA^TM^ microRNA ISH Optimization kit (FFPE) (Exiqon, Denmark). Briefly, after deparaffination and dehydration, specific miRNA probes were hybridized at 55 °C for 1 h. The samples were then washed several times, and incubated with blocking solution in a humidifying chamber for 15 min. The anti-DIG reagent was then applied for 60 min at room temperature. After washing, AP substrate was added and incubated at 30 °C for 2 h. The reaction was terminated by treatment with KTBT buffer(50 mM Tris at pH 7.5, 150 mM NaCl, 10 mM KCl, 1% Tween-20), and then counterstained with Nuclear Fast Red^TM^. All results were visualized on a BX50 microscope (Olympus, Tokyo, Japan).

### 4.6. Statistical Analysis

A total of 6 mice were selected by date and analyzed in vivo, and measurements were analyzed using one-way ANOVA. *p* values were marked with an asterisk, * *p* < 0.01, ** *p* < 0.001 and *** *p* < 0.0001.

## 5. Conclusions

Our results suggest that when the cochlear microstructures are damaged, miR-205, identified in stria vascularis, migrates through the blood vessels to organs or is randomly present in the blood. Therefore, in conditions of hearing impairment with ototoxic medications, the detection of circulating miR-205 in the blood can be used to determine the extent of hearing loss and inner ear damage and can be identified by simply performing a blood test before hearing impairment occurs. The precise mechanism of ototoxicity induced by aminoglycoside antibiotics has been suggested in various theories, but the mechanism induced by the kanamycin-furosemide combination established in this study is presumed to be a unique mechanism different from the existing mechanism. Thus, future studies will reveal its role in ototoxicity.

## Abbreviations

AKI	acute kidney injury
CEA	Carcino-Embryonic Antigen
CA-15-3	Carcinoma Antigen-15-3
dB	decibel
M	modiolus
miR	microRNA
OC	organ of Corti stria vascularis
SV	stria vascularis
TDM	Therapeutic drug monitoring
